# Prospective associations between psychosocial work factors and self-reported health: study of effect modification by gender, age, and occupation using the national French working conditions survey data

**DOI:** 10.1186/s12889-022-13773-x

**Published:** 2022-07-19

**Authors:** Isabelle Niedhammer, Laura Derouet-Gérault, Sandrine Bertrais

**Affiliations:** INSERM, Univ Angers, Univ Rennes, EHESP, Irset (Institut de recherche en santé, environnement et travail) - UMR_S 1085, ESTER Team, 28 rue Roger Amsler, CS 74521, 49045 ANGERS Cedex 01, France

**Keywords:** Psychosocial work factors, Self-reported health, Occupational exposures, Gender, Age, Occupation Effect modification

## Abstract

**Background:**

Prospective studies exploring the effects of psychosocial work factors on self-reported health (SRH) are lacking, especially those studying effect modifications. The objectives were to examine the prospective associations of these factors, and multiple exposures to these factors, with SRH in a national representative sample, and effect modifications by gender, age, and occupation.

**Methods:**

The prospective study relied on the three data collection waves (2013, 2016, and 2019) of the national French Working Conditions survey and was based on a sample of 15,971 employees, in good SRH at the beginning of the follow-up period. The occupational exposures were time-varying variables measured in 2013 and 2016, and included: 20 psychosocial work factors grouped into 5 broad domains, 4 exposures related to working time/hours and 4 physical-biomechanical-chemical exposures. The incidence of poor SRH three years later was the outcome. Discrete time Poisson regression models were performed using weighted data and with adjustment for gender, age, marital status, life events, and occupation.

**Results:**

Almost all the studied psychosocial work factors were predictive of poor SRH. Some physical-biomechanical-chemical exposures were found to predict poor SRH. Only rare effect modifications were observed according to gender, age, and occupation. Dose-response associations between multiple exposures and the incidence of poor SRH were observed for 4 among 5 domains of psychosocial work factors.

**Conclusions:**

Our study underlined the effects of psychosocial work factors, as well as multiple exposure effects, on the incidence of poor SRH. However, most of these effects were the same across population groups related to gender, age, and occupation.

**Supplementary Information:**

The online version contains supplementary material available at 10.1186/s12889-022-13773-x.

## Background

Psychosocial work factors defined by psychological and social exposures derived from the work organization and environment have been found as risk factors for various health outcomes, in particular mental disorders and cardiovascular diseases [1]. However, the data are lacking on the effect modifications by gender, age, and social position, although the rare previous studies suggested no or low effect modification [1].

Self-reported health (SRH) is recognized as a marker of general health and has been recommended for use by both WHO and EU commission [2]. SRH has also been shown to be a predictor of future morbidity and mortality [3–6]. A plethora of studies explored the associations between psychosocial work factors and SRH. However, the number of prospective studies, with clear chronological order between exposure and outcome, has been much lower [7–25]. Furthermore, the literature studied neither a large set of these factors nor the impact of multiple exposures, and only a few rare prospective studies explored effect modifications and suggested some effect modifications by gender [10, 22, 23].

The objectives of the present study were therefore to explore the prospective associations between occupational exposures, including a large set of psychosocial work factors and multiple exposures, and SRH in a nationally representative sample of the working population and potential effect modifications by gender, age, and social position.

## Methods

**Fig. 1:**
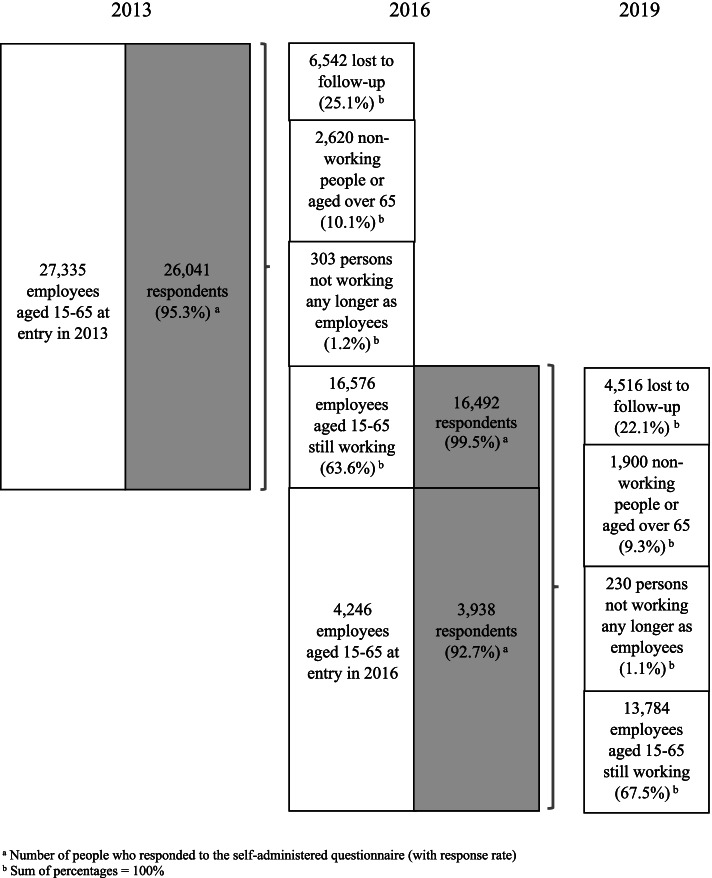
Flow chart

The study relied on the prospective data from the national French Working Conditions survey collected in 2013, 2016, and 2019 and set up by the French ministry of labour (DARES). The survey design is an open prospective cohort, i.e. people may have moved in and out the cohort during the follow-up. At each wave (2013, 2016, and 2019), the data were collected using a questionnaire administered by interviewer and a self-administered questionnaire. Three of our previous publications explored the cross-sectional associations between psychosocial work factors and various mental health outcomes (sleep problems, depression and anxiety, and suicide ideation) using the 2016 wave of the survey [26–28] and another one studied the prospective associations between psychosocial work factors and well-being using the two first waves (2013 and 2016) [29]. The flow chart presents sample size, response and attrition rates (Fig. 1). Two follow-up periods were used in the analyses: 2013–2016 and 2016–2019. The sample was restricted to 19,431 employees aged 15–65 at entry into the cohort who were working during a follow-up period.

SRH was chosen as the health outcome and was collected in the questionnaire in 2013, 2016, and 2019, using one single item (‘How would you rate your general health status?’) with 5 categories (very good, good, fair, poor, very poor) that was dichotomized into two groups: good (very good, good) and poor (fair, poor, very poor) SRH. This dichotomization was retained because the prevalence and incidence of poor/very poor SRH were very low (< 5 and < 3% respectively). Such a dichotomization was used previously in other studies among the French working population [30]. We studied the incidence of poor SRH three years later among the study sample of employees who were in good SRH at the beginning of a follow-up period.

Psychosocial work factors were collected in the questionnaire and the self-administered questionnaire in 2013 and 2016 and included twenty factors constructed using a total of 61 items. As no validated or recommended questionnaire was used in the survey, these items were selected because they were proxies of the items from the COPSOQ [31]. Some items, though not close to those of the COPSOQ, were considered relevant to the psychosocial work environment (changes at work, temporary employment). These 61 items allowed us to construct 20 factors, including 18 factors that were proxies of the factors of the COPSOQ. Our previous studies using the French Working Conditions survey data used the same strategy to construct psychosocial work factors [26–29]. All psychosocial work factors were grouped into 5 broad domains. Two other domains of occupational exposures were also studied: working time/hours (4 factors) and physical-biomechanical-chemical exposures (4 factors). The Appendix provides details on the content of all these exposures. Low and high exposure groups were defined using the initial coding for the factors based on one item and using the median of the total sample in 2013 as cut-off for the factors based on the sum of two or more items. Multiple exposures were constructed by summing the factors of the same domain. Multiple exposure was also calculated for all psychosocial work factors together, and defined using quartiles of the sum of all factors. The list of all domains and factors with corresponding number of items per each factor is provided in the Appendix (Additional file 1). The Appendix also provides median values to define low or high levels of exposure, and Cronbach’s alphas to assess internal consistency, as well as more information on the construction of the factors. Internal consistency was found to be satisfactory for most factors, but lower for some of them (for example predictability). Tetrachoric correlation coefficients were calculated between factors and are presented in Supplementary Table S1. Correlation coefficients were found to be low for most of these correlations. The study of multiple exposures to psychosocial work factors was a way to take the overlap, if any, between factors into account.

All statistical analyses were done using weighted data to correct for potential non-response and attrition bias and provide results that could be extrapolated to the target population. The prospective associations between occupational exposures, as time-varying variables (i.e. in 2013 or 2016), and the incidence of poor SRH three years later (i.e. in 2016 or 2019) were explored using discrete time Poisson regression models, with weighted data (option Stata pweight) and robust variance estimation (option Stata vce (robust)), and with adjustment for gender and the following time-varying covariates: age (4 10-year age groups), marital status (cohabiting or not), life events within the 3-year period (among 4 events: serious health problems of oneself or close family member, death of close family member, family conflict, and exposure to violence), and occupation (4 occupational groups) as a marker of social position. To explore effect modifications by gender, age, and occupation, multiplicative interaction terms were tested. Statistical trend tests were performed using orthogonal polynomial contrasts to study the dose-response associations between multiple exposures (i.e. the number of exposures) and the incidence of poor SRH. Multiple testing was corrected using False Discovery Rate (FDR), which is a method to control “the expected proportion of errors among the rejected hypotheses” [32]. All statistical analyses were performed using the following softwares: SAS version 9.04 and Stata version 15.0.

Sensitivity analyses included: (i) performing additional adjustment for chronic disease, (ii) performing additional adjustment for full/part time work, (iii) restricting the study sample to employees who stayed in the same job during follow-up, and (iv) performing additional adjustment for working time/hours and physical-biomechanical-chemical exposures in the study of multiple exposures to psychosocial work factors and SRH.

## Results

Among the sample of 19,431 employees, we retained for analysis only those with good SRH at the beginning of each follow-up period (in 2013 or 2016), i.e. 15,971 employees. Among them, 12,669 employees entered into the cohort in 2013 and were followed up from 2013 and 2016, including 6938 employees who continued to be followed up from 2016 to 2019. A total of 3302 employees entered into the cohort in 2016 and were followed up from 2016 to 2019. The incidence of poor SRH was 18.2% for the first period of follow-up (i.e. between 2013 and 2016 for those who entered into the cohort in 2013, and between 2016 and 2019 for those who entered in 2016) and 14.4% for the second period of follow-up (i.e. between 2016 and 2019). Descriptive statistics can be found in Supplementary Tables S2-S3.

Almost all psychosocial work factors were predictive of poor SRH, except cognitive demands and temporary employment (Table 1). After multiple testing correction according to FDR, one factor, emotional demands, was not predictive of poor SRH any longer. Biomechanical, fumes/dust and noise exposures predicted poor SRH.

**Table 1:** Prospective associations between occupational factors and the incidence of poor SRH among the study sample of 15,971 employees

	RR	95% CI			***p***-value
**Demands at work**					
Quantitative demands	1.36	1.21	–	1.53	**< 0.001**
Cognitive demands	1.11	0.99	–	1.25	0.086
Emotional demands	1.13	1.00	–	1.27	**0.044**‡
Demands for hiding emotions	1.24	1.10	–	1.39	**< 0.001**
**Work organization and job content**					
Low influence	1.14	1.02	–	1.28	**0.018**
Low degree of freedom	1.23	1.10	–	1.37	**< 0.001**
Low possibilities for development	1.19	1.06	–	1.33	**0.003**
Low meaning of work	1.32	1.19	–	1.47	**< 0.001**
**Interpersonal relations**					
Low predictability	1.13	1.01	–	1.26	**0.032**
Low role clarity	1.44	1.26	–	1.64	**< 0.001**
Role conflict	1.39	1.24	–	1.56	**< 0.001**
Low social support	1.22	1.09	–	1.38	**0.001**
Low sense of community	1.38	1.24	–	1.54	**< 0.001**
**Work–individual interface**					
Low job satisfaction	1.27	1.13	–	1.42	**< 0.001**
Work–family conflict	1.17	1.04	–	1.32	**0.010**
Job insecurity	1.20	1.05	–	1.37	**0.007**
High changes at work	1.34	1.19	–	1.50	**< 0.001**
Temporary employment	1.10	0.86	–	1.39	0.445
**Workplace violence**					
Internal violence	1.27	1.14	–	1.43	**< 0.001**
External violence	1.22	1.08	–	1.37	**0.001**
**Working time/hours**					
Long working hours (> 48 h/week)	0.84	0.67	–	1.04	0.113
Shift work	0.95	0.79	–	1.15	0.624
Unsocial work days	1.03	0.90	–	1.19	0.641
Night work	0.97	0.75	–	1.26	0.833
**Physical exposures**					
Biomechanical exposure	1.28	1.14	–	1.43	**< 0.001**
Fumes/dust	1.21	1.06	–	1.39	**0.006**
Toxic/dangerous products	1.05	0.92	–	1.20	0.476
Noise	1.23	1.07	–	1.41	**0.004**

RR: incidence rate ratio, CI: confidence interval

Each occupational factor was studied separately using discrete time Poisson regression models and weighted data, with adjustment for gender, age, marital status, life events, and occupation

Low or high exposure groups were defined using the initial coding for the factors based on one item (emotional demands, role clarity, work–family conflict, job insecurity, temporary employment) and using the median of the total sample in 2013 as cut-off for the factors based on the sum of two or more items

‡ *p* > 0.05 after correction for multiple testing (FDR)

28 tests were done, 1 or 2 would be significant at 5% even if the null hypotheses were true, and 21 were found to be significant (before correction for multiple testing, FDR, and 20 after correction)

A total of 6 effect modifications were observed: one by gender, one by age, and four by occupation (Table 2). Nevertheless, after correction for multiple testing, all the interactions were no longer significant, meaning no effect modification by gender, age, and occupation.

**Table 2:** Effect modifications by gender, age and occupation for the prospective associations between occupational factors and the incidence of poor SRH among the study sample of 15,971 employees

	RR	95% CI			***p***-value for interaction
Significant effect modifications by gender^1^					
**Job insecurity**					**0.019**‡
Men	**1.41**	**1.16**	**–**	**1.73**	
Women	1.03	0.87	–	1.23	
Significant effect modifications by age (years)^2^					
**Temporary employment**					**0.002**‡
< 30	0.67	0.45	–	1.01	
[30–40]	**1.73**	**1.18**	**–**	**2.55**	
[40–50]	1.23	0.82	–	1.85	
≥50	0.77	0.53	–	1.12	
Significant effect modifications by occupation^3^					
**High changes at work**					**0.039**‡
Managers - professionals	**1.52**	**1.15**	**–**	**2.01**	
Associate professionals - technicians	1.07	0.87	–	1.33	
Clerks - service workers	**1.60**	**1.33**	**–**	**1.93**	
Blue-collar workers	1.26	0.98	–	1.61	
**Shift work**					**0.006**‡
Managers - professionals	0.12	0.03	–	0.48	
Associate professionals - technicians	1.07	0.75	–	1.54	
Clerks - service workers	1.19	0.87	–	1.64	
Blue-collar workers	0.79	0.60	–	1.04	
**Biomechanical exposure**					**0.017**‡
Managers - professionals	1.05	0.75	–	1.46	
Associate professionals - technicians	1.05	0.86	–	1.28	
Clerks - service workers	**1.50**	**1.23**	**–**	**1.83**	
Blue-collar workers	**1.81**	**1.25**	**–**	**2.62**	
**Toxic/dangerous products**					**0.018**‡
Managers - professionals	0.95	0.64	–	1.42	
Associate professionals - technicians	1.03	0.84	–	1.27	
Clerks - service workers	**1.40**	**1.13**	**–**	**1.72**	
Blue-collar workers	0.83	0.64	–	1.06	

*RR* incidence rate ratio, *CI* confidence interval

Each occupational factor was studied separately using discrete time Poisson regression models and weighted data

^*1*^Adjusted for age, marital status, life events, and occupation

^*2*^Adjusted for gender, marital status, life events, and occupation

^3^Adjusted for gender, age, marital status, and life events

‡ *p* > 0.05 after correction for multiple testing (FDR)

84 tests were done, 4 or 5 would be significant at 5% even if the null hypotheses were true, and 6 were found to be significant (before correction for multiple testing, FDR, and 0 after correction)

**Fig. 2:**
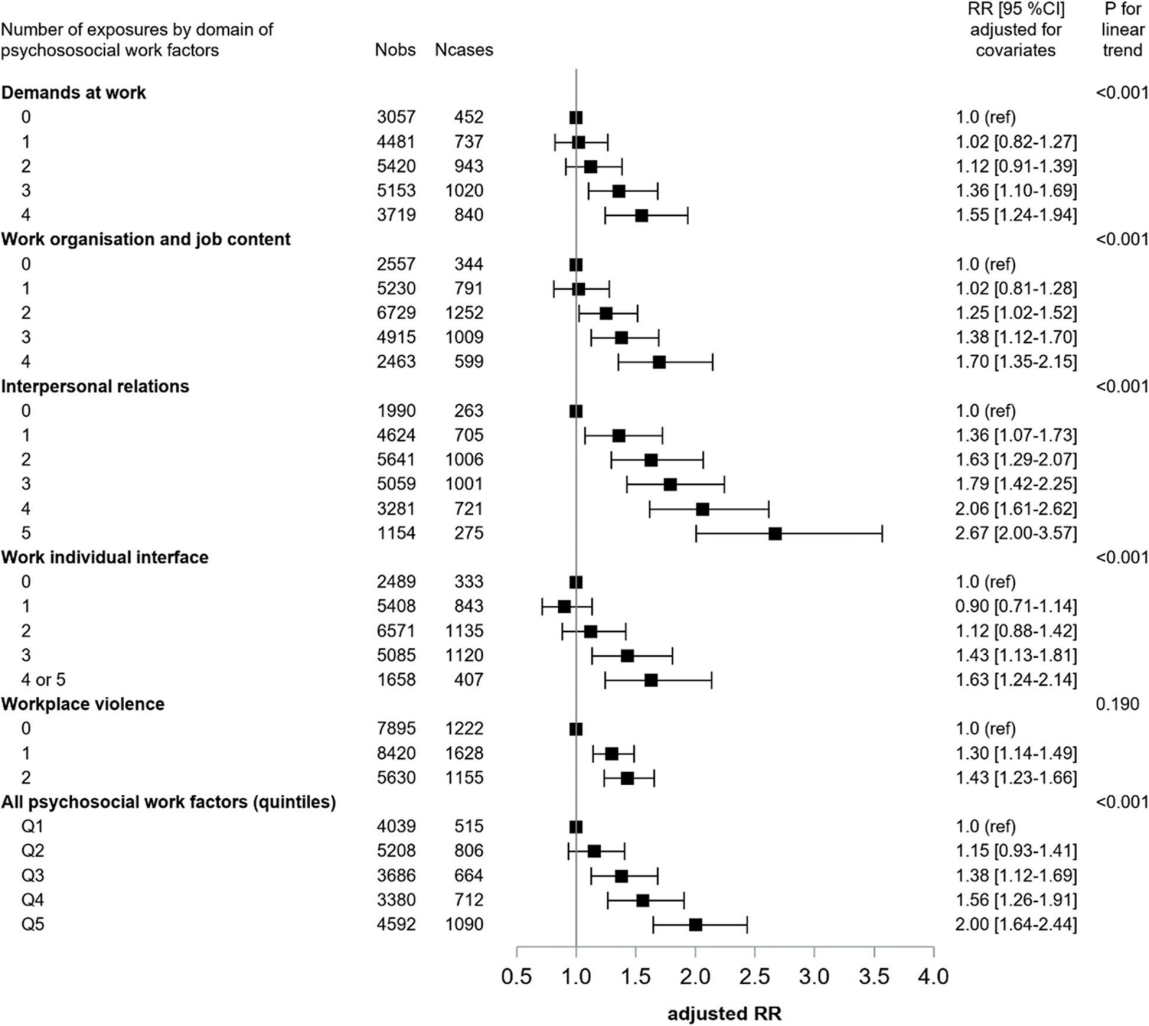
Prospective associations between multiple exposures to psychosocial work factors and the incidence of poor SRH among the study sample of 15,971 employees

Statistical trend tests were significant for all measures of multiple exposure to psychosocial work factors for all domains (except violence) and for all psychosocial work factors together (Fig. 2), suggesting dose-response prospective associations between the number of exposures and the incidence of poor SRH. The associations between multiple exposures to physical-biomechanical-chemical exposures and working time/hours factors and SRH were not significant (not shown).

Sensitivity analyses showed that the results were unchanged after additional adjustment for chronic disease or full/part time work. Restricting the study sample to the employees who remained in the same job during follow-up provided the same results, although two psychosocial work factors were no longer predictive of poor SRH: low influence and work-family conflict. Additional adjustment for working time/hours and physical-biomechanical-chemical exposures in the study of multiple exposures to psychosocial work factors and SRH did not change the results.

## Discussion

This study showed that almost all the 20 studied psychosocial work factors were predictive of poor SRH. There was however only rare effect modifications by gender, age, and occupation, suggesting that most of the exposure-outcome associations were the same according to these variables. Dose-response associations were found between multiple exposures to psychosocial work factors and the incidence of poor SRH.

Previous prospective studies reported associations of job insecurity [7–9, 15, 21, 24], high workpace or psychological demands [8–10, 19], low influence and possibilities for development [8–10], low social support [8–10, 14, 19, 22], and conflict or violence [9, 13] and SRH, in agreement with our results. Our study explored a large set and detailed measures of psychosocial work factors and showed that additional factors were predictive of poor SRH, such as role stressors, work-family conflict, changes at work, etc., associations that were not reported before. Few previous studies explored other occupational exposures in association with SRH prospectively, and found associations for long working hours [11], shift work [11], and physical or ergonomic demands [7–9, 19], in line with our results for physical-biomechanical-chemical exposures, but not for working time/hours. Three prospective studies [10, 22, 23] explored effect modifications by gender, age, and/or social position, but only effect modifications by gender were found. In the studies by Schmidt et al. [22] and by Stadin et al. [23], lack of supportive leadership behavior and ICT (information and communication technology) demands at work predicted poor SRH among men and not among women. No effect modification by age and social position (occupation) was reported previously. Our results echoed the literature in the sense that almost no effect modification was found. As both the test of multiplicative interaction terms and correction for multiple testing may be conservative, the rare interactions observed in our study may be of interest. In particular, the interaction between gender and job insecurity suggested that job insecurity might predict poor SRH among men only, referring to the breadwinner model. Similar findings were found in the literature for mental health outcomes [33–37]. There was no previous study examining multiple exposures in association with SRH, consequently our study may be the first one to suggest the deleterious effects of multiple exposures to psychosocial work factors on SRH.

Strengths of our study deserve to be presented. Our study had a prospective design with clear chronological order between exposure and outcome. Response and follow-up rates were satisfactory and did not lead to major response and attribution bias. Indeed, the comparison between respondents and people lost to follow-up did not show major differences (although some of these differences were statistically significant) in the studied covariates, occupational exposures and SRH (Supplementary Table S4). Furthermore, we used weights to correct for these potential biases. It should be noticed that when the statistical analyses were done without weighted data, the results were the same as well as our conclusions. The study sample was large and nationally representative. We used time-varying exposure measures and studied a large set of exposures and multiple exposures. We tested effect modifications by gender, age, and occupation, which has been very seldom in the literature. We used SRH as a recognized general health outcome. Our models were adjusted for relevant covariates. We performed a correction for multiple testing. Nevertheless, correction for multiple testing may be a conservative approach, especially in the case of high number of tests, and rare and low true associations. This was not the case for the study of the associations between psychosocial work factors and SRH (these associations were almost all significant), but this was the case for the study of interactions (only some rare interactions were found). Consequently, some interactions might truly be significant. The issue of whether or not correcting for multiple testing has been debated at length in the literature with pros and cons [38, 39]. This is why we presented all our results before and after correction for multiple testing. Sensitivity analyses confirmed our results.

Some limitations may nevertheless be underlined. A healthy worker effect may be low as we found no major difference in the results of the main analysis and the sensitivity analysis restricted to the employees who stayed in the same job during follow-up. There was a potential reporting bias, as both exposures and outcome were self-reported. In addition, this bias may be related to personal factors such as personal coping pattern or response style, which were not available and not controlled for. Nevertheless, SRH is by definition a perception. Furthermore, reporting bias may be low given the prospective design of the study. We constructed proxies of the factors of the COPSOQ, as the recommended COPSOQ questionnaire was not used, which may have led to imprecision and misclassification. Furthermore, imprecision and misclassification may be higher for the factors that were based on a lower number of items and/or displayed lower internal consistency. Information was also lacking in the changes in the exposures between two waves of data collection, leading to lack of precision, misclassification, and bias towards the null hypothesis. Some psychosocial work factors (organization injustice for example) and covariates (social support outside the workplace for example) may be missing. We tested multiplicative interaction terms to study effect modifications, and this approach is considered conservative, compared to other approaches (additive interaction for example). Nevertheless, the test of additive gender-related interactions in our study provided two significant interactions only for job insecurity –that was also found with the test of multiplicative interactions- and for noise. Consequently, there was no major differences between multiplicative and additive interactions. This might be explained by a high statistical power related to large sample size.

To conclude, psychosocial work factors were found to predict the incidence of poor SRH. Almost all the associations of these factors with SRH were the same across gender, age, and occupation groups. Multiple exposure to these factors displayed dose-response associations with poor SRH. Comprehensive prevention oriented towards the psychosocial work environment is likely to improve SRH in the whole working population. More attention should be given to multiple exposures to psychosocial work factors.

## Supplementary Information

**Additional file 1.**

**Additional file 2.**

## Data Availability

The dataset used and analysed during the current study are available from the corresponding author on reasonable request.
