# Supplementary material for: Prospective associations between psychosocial work factors and self-reported health: study of effect modification by gender, age, and occupation using the national French working conditions survey data
Source: BMC Public Health. 2022 Jul 19;22:1389. doi: 10.1186/s12889-022-13773-x (PMC9295500; doi:10.1186/s12889-022-13773-x)
Supplement: Supplementary file 1 — Additional file 1. [file 12889_2022_13773_MOESM1_ESM.docx]

**Appendix** List of domains and factors with corresponding number of items, Cronbach’s alpha, and median cut-off per each factor

| Domains (number of factors) | | Factors (number of items) | Cronbach’s alpha | Median cut-off for low/high exposure |
| --- | --- | --- | --- | --- |
| **PSYCHOSOCIAL WORK FACTORS** | |  |  |  |
| **Demands at work (4 factors)** | |  |  |  |
|  | | Quantitative demands (7 items) | 0.72 | 1.99 |
|  | | Cognitive demands (3 items) | 0.49 | 1.66 |
|  | | Emotional demands (1 binary item) | *-* | *-* |
|  | | Demands for hiding emotions (2 items) | 0.63 | 0.66 |
| **Work organization and job content**  **(4 factors)** | |  |  |  |
|  | | Influence (5 items) | 0.47 | 1.66 |
|  | | Degree of freedom (3 items) | 0.54 | 0.50 |
|  | | Possibilities for development (3 items) | 0.55 | 0.66 |
|  | | Meaning of work (3 items) | 0.61 | 1.32 |
| **Interpersonal relations (5 factors)** | |  |  |  |
|  | | Predictability (2 items) | 0.32 | 0.66 |
|  | | Role clarity (1 binary item) | *-* | *-* |
|  | | Role conflict (3 items) | 0.57 | 0.99 |
|  | | Social support (7 items) | 0.60 | 1.99 |
|  | | Sense of community (3 items) | 0.47 | 0.99 |
| **Work–individual interface (5 factors)** | |  |  |  |
|  | | Job satisfaction (3 items) | 0.64 | 1.41 |
|  | | Work–family conflict (1 item) | *-* | 0.33 |
|  | | Job insecurity (1 binary item) | *-* | *-* |
|  | | Changes at work (2 items) | 0.58 | 0.66 |
|  | | Temporary employment (1 binary item) | *-* | *-* |
| **Workplace violence (2 factors)** | |  |  |  |
|  | | Internal violence (6 items) | 0.61 | 1.00 |
|  | | External violence (4 items) | 0.59 | 1.00 |
| **OTHER OCCUPATIONAL EXPOSURES** | |  |  |  |
| **Working time/hours (4 factors)** | |  |  |  |
|  | | Long working hours (>48h/week) (1 binary item) | *-* | *-* |
|  | | Shift work (1 binary item) | *-* | *-* |
|  | | Unsocial work days (>40/year) (1 binary item) | *-* | *-* |
|  | | Night work (>50/year) (1 binary item) | *-* | *-* |
| **Physical-biomechanical-chemical exposures (4 factors)** | |  |  |  |
|  | | Biomechanical exposure (6 items) | 0.77 | 2.33 |
|  | | Fumes/dust (1 binary item) | *-* | *-* |
|  | | Toxic/dangerous products (1 binary item) | *-* | *-* |
|  | | Noise (1 binary item) | *-* | *-* |
| Each factor, assessed using more than one item, was constructed by summing all the items of the factor (beforehand each item was coded on a scale from 0 to 1, with 0 indicating no exposure and 1 exposure). Thus, each factor had a score ranging from 0 to a maximum value that corresponded to the number of items, and was then dichotomized at the median of the distribution in the total sample to classify workers into low (< median) or high (≥ median) exposure groups. The results were based on the analysis of the 2013 data among the 26,041 employees aged 15-65 who responded to the self-administered questionnaire (see flow chart in Figure 1). | | | |  |
