# Supplementary material for: Prospective associations between psychosocial work factors and self-reported health: study of effect modification by gender, age, and occupation using the national French working conditions survey data
Source: BMC Public Health. 2022 Jul 19;22:1389. doi: 10.1186/s12889-022-13773-x (PMC9295500; doi:10.1186/s12889-022-13773-x)
Supplement: Supplementary file 2 — Additional file 2. [file 12889_2022_13773_MOESM2_ESM.docx]

**Supplementary Table S1** Tetrachoric correlation coefficients between exposures to psychosocial work factors

|  | Quantitative demands | Cognitive demands | Emotional demands | Demands for hiding emotions | Influence | Degree of freedom | Possibilities for development | Meaning of work | Predictability | Role clarity | Role conflict | Social support | Sense of community | Job satisfaction | Work–family conflict | Job insecurity | Changes at work | Temporary employment | Internal violence | External violence |
| --- | --- | --- | --- | --- | --- | --- | --- | --- | --- | --- | --- | --- | --- | --- | --- | --- | --- | --- | --- | --- |
| Quantitative demands | - | 0.583 | 0.286 | 0.409 | 0.094 | 0.218 | -0.007 | 0.136 | 0.056 | 0.485 | 0.494 | 0.324 | 0.180 | 0.353 | 0.318 | 0.133 | 0.530 | -0.187 | 0.437 | 0.347 |
| Cognitive demands |  | - | 0.245 | 0.280 | -0.102 | -0.069 | -0.094 | 0.061 | 0.126 | 0.359 | 0.481 | 0.237 | 0.023 | 0.202 | 0.226 | 0.019 | 0.486 | -0.304 | 0.389 | 0.284 |
| Emotional demands |  |  | - | 0.335 | 0.056 | 0.209 | -0.031 | -0.037 | -0.077 | 0.184 | 0.255 | 0.197 | 0.029 | 0.202 | 0.115 | 0.009 | 0.266 | -0.123 | 0.283 | 0.503 |
| Demands for hiding emotions |  |  |  | - | 0.160 | 0.312 | 0.270 | 0.204 | -0.034 | 0.315 | 0.404 | 0.357 | 0.345 | 0.366 | 0.238 | 0.195 | 0.461 | -0.083 | 0.438 | 0.358 |
| Influence |  |  |  |  | - | 0.356 | 0.185 | 0.185 | 0.018 | 0.035 | 0.140 | 0.128 | 0.127 | 0.178 | 0.161 | 0.169 | 0.121 | 0.183 | 0.151 | 0.061 |
| Degree of freedom |  |  |  |  |  | - | 0.206 | 0.074 | 0.004 | 0.135 | 0.160 | 0.215 | 0.175 | 0.253 | 0.298 | 0.174 | 0.132 | 0.164 | 0.149 | 0.187 |
| Possibilities for development |  |  |  |  |  |  | - | 0.412 | -0.027 | 0.220 | 0.197 | 0.304 | 0.454 | 0.419 | 0.099 | 0.266 | 0.162 | 0.015 | 0.211 | 0.028 |
| Meaning of work |  |  |  |  |  |  |  | - | 0.102 | 0.239 | 0.257 | 0.241 | 0.475 | 0.325 | 0.152 | 0.198 | 0.201 | -0.003 | 0.241 | 0.015 |
| Predictability |  |  |  |  |  |  |  |  | - | 0.139 | 0.138 | 0.040 | 0.041 | 0.033 | 0.115 | 0.124 | 0.164 | 0.090 | 0.058 | -0.010 |
| Role clarity |  |  |  |  |  |  |  |  |  | - | 0.520 | 0.405 | 0.292 | 0.408 | 0.205 | 0.190 | 0.461 | -0.149 | 0.409 | 0.204 |
| Role conflict |  |  |  |  |  |  |  |  |  |  | - | 0.418 | 0.265 | 0.413 | 0.207 | 0.189 | 0.538 | -0.140 | 0.536 | 0.291 |
| Social support |  |  |  |  |  |  |  |  |  |  |  | - | 0.411 | 0.462 | 0.224 | 0.143 | 0.341 | -0.217 | 0.475 | 0.199 |
| Sense of community |  |  |  |  |  |  |  |  |  |  |  |  | - | 0.420 | 0.171 | 0.264 | 0.231 | -0.023 | 0.382 | 0.039 |
| Job satisfaction |  |  |  |  |  |  |  |  |  |  |  |  |  | - | 0.226 | 0.281 | 0.365 | -0.096 | 0.467 | 0.228 |
| Work–family conflict |  |  |  |  |  |  |  |  |  |  |  |  |  |  | - | 0.161 | 0.288 | -0.062 | 0.226 | 0.158 |
| Job insecurity |  |  |  |  |  |  |  |  |  |  |  |  |  |  |  | - | 0.205 | 0.386 | 0.213 | 0.038 |
| Changes at work |  |  |  |  |  |  |  |  |  |  |  |  |  |  |  |  | - | -0.140 | 0.438 | 0.293 |
| Temporary employment |  |  |  |  |  |  |  |  |  |  |  |  |  |  |  |  |  | - | -0.164 | -0.158 |
| Internal violence |  |  |  |  |  |  |  |  |  |  |  |  |  |  |  |  |  |  | - | 0.359 |
| External violence |  |  |  |  |  |  |  |  |  |  |  |  |  |  |  |  |  |  |  | - |

The results were based on binary variables and on the analysis of the 2013 weighted data among 26,041 employees aged 15-65 years who responded to the self-administered questionnaire (see flow chart in Figure 1).

Supplementary Table S2 Description of the study sample of 15,971 employees with good SRH at the beginning of each follow-up period

|  | **First follow-up period**  **(15,971 participants)** | | **Second follow-up period**  **(6938 participants**) | |
| --- | --- | --- | --- | --- |
|  | n^a^ | w%^b^ | n^a^ | w%^b^ |
| **Period of follow-up** |  |  |  |  |
| 2013-2016 | 12669 | 55.4 | - | - |
| 2016-2019 | 3302 | 44.6 | 6938 | 100.0 |
| **Gender** |  |  |  |  |
| Men | 6983 | 50.9 | 3114 | 53.5 |
| Women | 8988 | 49.1 | 3824 | 46.5 |
| **Age (years)^c^** |  |  |  |  |
| <30 | 2366 | 22.2 | 475 | 11.9 |
| [30-40[ | 4327 | 29.1 | 1835 | 31.1 |
| [40-50[ | 5415 | 29.5 | 2639 | 33.6 |
| ≥50 | 3863 | 19.2 | 1989 | 23.3 |
| **Marital status^c^** |  |  |  |  |
| Living with a partner | 12439 | 74.9 | 5584 | 80.5 |
| Living alone | 3532 | 25.1 | 1354 | 19.5 |
| **Occupation^c^** |  |  |  |  |
| Managers, professionals | 3032 | 20.8 | 1419 | 21.9 |
| Associate professionals, technicians | 5238 | 29.5 | 2344 | 30.3 |
| Clerks, service workers | 5028 | 29.2 | 2103 | 28.6 |
| Blue-collar workers | 2653 | 20.5 | 1008 | 19.3 |
| **Life events within the 3-year follow-up period** |  |  |  |  |
| None | 7491 | 50.5 | 3113 | 48.9 |
| 1 | 4926 | 31.2 | 2212 | 31.8 |
| ≥ 2 | 2879 | 18.4 | 1440 | 19.3 |
| **Incidence of poor SRH** | 3144 | 18.2 | 1046 | 14.4 |

^a^ Unweighted number

^b^ Weighted percentage

^c^ At the beginning of the follow-up period

**Supplementary Table S3** Description of the exposure to occupational factors among the study sample of 15,971 employees with good SRH at the beginning of each follow-up period

|  | **First follow-up period**  **(15,971 participants)** | | **Second follow-up period**  **(6938 participants**) | |
| --- | --- | --- | --- | --- |
| **Occupational factors^a^** | n^b^ | w%^c^ | n^b^ | w%^c^ |
| **Demands at work** |  |  |  |  |
| Quantitative demands | 7864 | 46.6 | 3393 | 46.6 |
| Cognitive demands | 8050 | 48.1 | 3527 | 50.0 |
| Emotional demands | 8598 | 45.6 | 3887 | 47.2 |
| Demands for hiding emotions | 8790 | 50.2 | 3495 | 46.1 |
| **Work organization and job content** |  |  |  |  |
| Low influence | 7968 | 49.7 | 3279 | 47.1 |
| Low degree of freedom | 8515 | 50.9 | 3541 | 46.7 |
| Low possibilities for development | 8149 | 51.3 | 3313 | 48.4 |
| Low meaning of work | 7508 | 46.8 | 3034 | 45.1 |
| **Interpersonal relations** |  |  |  |  |
| Low predictability | 8592 | 57.2 | 3540 | 54.4 |
| Low role clarity | 3008 | 18.5 | 1297 | 17.7 |
| Role conflict | 8035 | 48.8 | 3422 | 48.4 |
| Low social support | 9706 | 56.4 | 4118 | 56.2 |
| Low sense of community | 7509 | 46.9 | 3240 | 47.0 |
| **Work–individual interface** |  |  |  |  |
| Low job satisfaction | 8535 | 49.6 | 3445 | 47.8 |
| Work–family conflict | 10022 | 60.5 | 4199 | 60.6 |
| Job insecurity | 3009 | 22.2 | 1048 | 17.7 |
| High changes at work | 8040 | 47.2 | 3528 | 48.7 |
| Temporary employment | 1467 | 13.7 | 324 | 7.7 |
| **Workplace violence** |  |  |  |  |
| Internal violence | 7648 | 44.8 | 3201 | 43.4 |
| External violence | 6678 | 36.7 | 2859 | 36.6 |
| **Working time/hours** |  |  |  |  |
| Long working hours (>48h/week) | 1232 | 8.9 | 546 | 8.8 |
| Shift work | 1372 | 7.7 | 521 | 7.2 |
| Unsocial work days | 2759 | 16.8 | 1135 | 16.8 |
| Night work | 1066 | 5.8 | 448 | 5.9 |
| **Physical exposures** |  |  |  |  |
| Biomechanical exposure | 8388 | 51.0 | 3443 | 47.7 |
| Fumes/dust | 3998 | 27.8 | 1690 | 27.9 |
| Toxic/dangerous products | 4847 | 28.9 | 2071 | 28.8 |
| Noise | 2265 | 15.5 | 932 | 15.2 |

^a^ Low or high exposure groups were defined using the initial coding for the factors based on one item (emotional demands, role clarity, work–family conflict, job insecurity, temporary employment) and using the median of the total sample in 2013 as cut-off for the factors based on the sum of two or more items – All the occupational factors were measured at the beginning of each follow-up period

^b^ Unweighted number

^c^ Weighted percentage

**Supplementary Table S4** Comparison between respondents and lost to follow-up for each 3-year period

|  | **From 2013 to 2016 (N=26,041)** | | | | | | |  | **From 2016 to 2019 (N=20,430)** | | | | | | |
| --- | --- | --- | --- | --- | --- | --- | --- | --- | --- | --- | --- | --- | --- | --- | --- |
|  | **Lost to follow-up**  **(n=6,542)** | |  | **Respondents**  **(n=19,499)** | |  | **p-value^c^** |  | **Lost to follow-up**  **(n=4,516)** | |  | **Respondents**  **(n=15,914)** | |  | **p-value^c^** |
|  | **n^a^** | **w%^b^** |  | **n^a^** | **w%^b^** |  |  |  | **n^a^** | **w%^b^** |  | **n^a^** | **w%^b^** |  |  |
| **COVARIATES** | | | | | | | | | | | | | | | |
| **Gender** |  |  |  |  |  |  |  |  |  |  |  |  |  |  |  |
| Men | 2998 | 53.9 |  | 8187 | 48.4 |  | <0.001 |  | 1994 | 52.9 |  | 6585 | 49.1 |  | 0.024 |
| Women | 3544 | 46.1 |  | 11312 | 51.6 |  |  |  | 2522 | 47.1 |  | 9329 | 50.9 |  |  |
| **Age (years)** |  |  |  |  |  |  |  |  |  |  |  |  |  |  |  |
| <30 | 1268 | 26.5 |  | 2109 | 15.7 |  | <0.001 |  | 775 | 26.7 |  | 1528 | 15.6 |  | <0.001 |
| [30-40[ | 1829 | 27.7 |  | 4746 | 23.9 |  |  |  | 960 | 23.6 |  | 3377 | 24.9 |  |  |
| [40-50[ | 1825 | 25.5 |  | 6404 | 30.5 |  |  |  | 1384 | 24.9 |  | 5235 | 29.7 |  |  |
| ≥50 | 1620 | 20.3 |  | 6240 | 30.0 |  |  |  | 1397 | 24.9 |  | 5774 | 29.9 |  |  |
| **Marital status** |  |  |  |  |  |  |  |  |  |  |  |  |  |  |  |
| Living with a partner | 4659 | 69.2 |  | 14995 | 76.3 |  | <.0001 |  | 3290 | 71.6 |  | 12386 | 75.4 |  | 0.012 |
| Living alone | 1883 | 30.8 |  | 4504 | 23.7 |  |  |  | 1225 | 28.4 |  | 3528 | 24.6 |  |  |
| **Occupation** |  |  |  |  |  |  |  |  |  |  |  |  |  |  |  |
| Managers, professionals | 1109 | 19.8 |  | 3334 | 18.7 |  | 0.318 |  | 797 | 17.4 |  | 2919 | 19.1 |  | 0.022 |
| Associate professionals, technicians | 1881 | 24.8 |  | 6001 | 26.5 |  |  |  | 1304 | 24.5 |  | 5076 | 27.8 |  |  |
| Clerks, service workers | 2301 | 31.6 |  | 6606 | 30.9 |  |  |  | 1539 | 32.8 |  | 5215 | 30.8 |  |  |
| Blue-collar workers | 1247 | 23.8 |  | 3547 | 23.9 |  |  |  | 843 | 25.3 |  | 2589 | 22.3 |  |  |
| **OCCUPATIONAL EXPOSURES** | | | | | | | | | | | | | | | |
| **Demands at work** |  |  |  |  |  |  |  |  |  |  |  |  |  |  |  |
| Quantitative demands | 3316 | 49.1 |  | 10000 | 49.8 |  | 0.552 |  | 2271 | 47.7 |  | 8085 | 48.3 |  | 0.723 |
| Cognitive demands | 3286 | 49.5 |  | 9863 | 48.6 |  | 0.482 |  | 2254 | 47.2 |  | 8219 | 48.6 |  | 0.419 |
| Emotional demands | 3334 | 42.5 |  | 10606 | 46.5 |  | <0.001 |  | 2416 | 44.6 |  | 9095 | 48.2 |  | 0.029 |
| Demands for hiding emotions | 3865 | 56.4 |  | 11661 | 57.0 |  | 0.605 |  | 2435 | 52.4 |  | 8880 | 52.7 |  | 0.845 |
| **Work organization and job content** |  |  |  |  |  |  |  |  |  |  |  |  |  |  |  |
| Low influence | 3640 | 54.2 |  | 10056 | 50.9 |  | 0.006 |  | 2384 | 57.0 |  | 7850 | 50.4 |  | <0.001 |
| Low degree of freedom | 3807 | 55.4 |  | 10771 | 52.4 |  | 0.015 |  | 2053 | 46.0 |  | 6941 | 42.8 |  | 0.054 |
| Low possibilities for development | 3730 | 58.0 |  | 10871 | 58.4 |  | 0.708 |  | 2491 | 57.0 |  | 8479 | 54.5 |  | 0.124 |
| Low meaning of work | 3227 | 51.0 |  | 9702 | 52.0 |  | 0.391 |  | 2156 | 49.5 |  | 7572 | 48.2 |  | 0.450 |
| **Interpersonal relations** |  |  |  |  |  |  |  |  |  |  |  |  |  |  |  |
| Low predictability | 3811 | 60.6 |  | 10392 | 55.6 |  | <0.001 |  | 2439 | 56.4 |  | 8207 | 55.8 |  | 0.743 |
| Low role clarity | 1279 | 18.8 |  | 4058 | 20.3 |  | 0.124 |  | 935 | 18.5 |  | 3383 | 20.3 |  | 0.141 |
| Role conflict | 3403 | 52.6 |  | 10183 | 51.1 |  | 0.206 |  | 2406 | 51.2 |  | 8418 | 51.4 |  | 0.900 |
| Low social support | 3464 | 50.5 |  | 10547 | 51.7 |  | 0.302 |  | 2382 | 49.2 |  | 8489 | 49.8 |  | 0.691 |
| Low sense of community | 3414 | 51.8 |  | 10127 | 53.0 |  | 0.351 |  | 2351 | 51.3 |  | 8207 | 51.6 |  | 0.880 |
| **Work–individual interface** |  |  |  |  |  |  |  |  |  |  |  |  |  |  |  |
| Low job satisfaction | 3562 | 50.8 |  | 10809 | 53.5 |  | 0.024 |  | 2461 | 51.1 |  | 8707 | 51.5 |  | 0.782 |
| Work–family conflict | 4341 | 66.0 |  | 12613 | 63.9 |  | 0.071 |  | 2929 | 64.0 |  | 10021 | 61.1 |  | 0.074 |
| Job insecurity | 1424 | 25.3 |  | 4147 | 25.3 |  | 0.989 |  | 974 | 25.2 |  | 3037 | 24.6 |  | 0.675 |
| High changes at work | 3362 | 50.0 |  | 10404 | 51.6 |  | 0.195 |  | 2359 | 48.1 |  | 8703 | 51.1 |  | 0.077 |
| Temporary employment | 897 | 17.5 |  | 1870 | 12.6 |  | <0.001 |  | 467 | 17.8 |  | 1068 | 14.5 |  | 0.026 |
| **Workplace violence** |  |  |  |  |  |  |  |  |  |  |  |  |  |  |  |
| Internal violence | 3295 | 48.6 |  | 9962 | 48.8 |  | 0.827 |  | 2188 | 46.3 |  | 8039 | 47.7 |  | 0.407 |
| External violence | 2797 | 38.1 |  | 8312 | 38.0 |  | 0.934 |  | 1887 | 38.4 |  | 6776 | 37.6 |  | 0.606 |
| **Working time/hours** |  |  |  |  |  |  |  |  |  |  |  |  |  |  |  |
| Long working hours (>48h/week) | 593 | 12.2 |  | 1409 | 8.2 |  | <0.001 |  | 347 | 8.9 |  | 1183 | 8.3 |  | 0.451 |
| Shift work | 572 | 7.6 |  | 1682 | 8.0 |  | 0.582 |  | 356 | 8.2 |  | 1253 | 7.9 |  | 0.725 |
| Unsocial work days (>40/year) | 1290 | 19.4 |  | 3468 | 17.6 |  | 0.055 |  | 837 | 19.7 |  | 2721 | 16.8 |  | 0.021 |
| Night work (>50/year) | 416 | 5.9 |  | 1251 | 5.8 |  | 0.832 |  | 288 | 6.5 |  | 979 | 5.7 |  | 0.336 |
| **Physical exposures** |  |  |  |  |  |  |  |  |  |  |  |  |  |  |  |
| Biomechanical exposure | 3197 | 48.6 |  | 9093 | 46.0 |  | 0.033 |  | 2167 | 48.5 |  | 7304 | 45.3 |  | 0.053 |
| Fumes/dust | 1979 | 31.5 |  | 5317 | 30.9 |  | 0.573 |  | 1275 | 33.3 |  | 4287 | 29.5 |  | 0.016 |
| Toxic/dangerous products | 2017 | 29.5 |  | 6078 | 30.0 |  | 0.653 |  | 1406 | 30.0 |  | 4910 | 29.5 |  | 0.754 |
| Noise | 1073 | 17.6 |  | 3115 | 18.5 |  | 0.348 |  | 782 | 19.3 |  | 2636 | 18.2 |  | 0.403 |
| **Sel-reported health** |  |  |  |  |  |  |  |  |  |  |  |  |  |  |  |
| Good | 4961 | 78.8 |  | 14380 | 74.9 |  | 0.001 |  | 3229 | 75.8 |  | 11455 | 74.2 |  | 0.247 |
| Poor | 1571 | 21.2 |  | 5078 | 25.1 |  |  |  | 1282 | 24.2 |  | 4444 | 25.8 |  |  |

^a^ Unweighted number

^b^ Weighted percentage

^c^ P-value from Rao-Scott chi-square test

Comparison was done using the variables at the beginning of the 3-year period
